# Justice in Health? Studying the Role of Legal Support in a Culturally Responsive Mental Health Service in Australia

**DOI:** 10.1177/10497323251315435

**Published:** 2025-04-02

**Authors:** Stefanie Plage, Rebecca E. Olson, Nathalia Costa, Karime Mescouto, Sameera Suleman, Asma Zulfiqar, Jenny Setchell, Rita Prasad-ildes

**Affiliations:** 1 ARC Centre of Excellence for Children and Families over the Life Course at the School of Social Science, 1974 The University of Queensland, St Lucia, QLD, Australia; 2 School of Social Science, 144911 The University of Queensland, St Lucia, QLD, Australia; 3 Clinical Trial Capability Team, 1974 The University of Queensland, St Lucia, QLD, Australia; 4 RECOVER Injury Research Centre, 117664 The University of Queensland, St Lucia, QLD, Australia; 5 School of Health and Rehabilitation Sciences, 104826 The University of Queensland, St Lucia, QLD, Australia; 6 World Wellness Group, Brisbane, QLD, Australia; 7 School of Social and Political Sciences, 228563 University of Melbourne, Melbourne, VIC, Australia; 8 613101 Institute for Urban Indigenous Health, Windsor, QLD, Australia

**Keywords:** Health Justice Partnerships, culturally and racially marginalized, Australia, mental health, culturally responsive qualitative research

## Abstract

Health Justice Partnerships (HJPs) are collaborations across law, health, and social care seeking more equitable health outcomes. This article aims to explore an HJP embedded within a culturally responsive mental health service in Australia for people who are culturally and racially marginalized (CARM). We draw on data produced for an evaluation of this service between August and November 2022 to conduct a reflexive thematic analysis. Thinking conceptually with the social determinants of health and intersectionality operationalized as structural, political, and representational, we present findings from individual and group interviews with 16 service users and 37 service providers. First, we describe the variety of legal issues service providers and service users encounter and how they affect opportunities for good health. Second, we provide insights into how care coordination across practitioners from different sectors and professions takes place to support service users. Third, we identify service principles and values that inform practices of integrated and culturally responsive care. We tie these insights together to demonstrate how multiple social categories flow together in the experiences of people from CARM communities in Western, White normative, and/or settler colonial societies. People confront built-in legal issues, for example, related to immigration legal status, welfare, housing, employment, or family, that affect mental health. Health, legal, and social systems have the dual capacity to capture people from CARM backgrounds in relations of care as well as oppression. We offer methodological reflections on studying these dynamics through culturally responsive qualitative research and discuss implications for culturally responsive HJP practice.

## Introduction

Health Justice Partnerships (HJPs) are collaborations across law, health, and social care aiming for more equitable health outcomes. Called Medical-Legal Partnerships in the United States, HJPs are hailed as achieving health outcomes through legal support (Curran, 2017; Genn, 2019; Martinez et al., 2017; Tobin-Tyler et al., 2023). In Australia, these partnerships are positioned as “pioneering collaborations between legal and healthcare professionals, strategically integrating legal assistance into healthcare services” to alleviate the inequitable conditions facing people experiencing socio-economic disadvantage (Lewis et al., 2019; World Wellness Group (WWG) and Caxton Legal Centre (CLC), 2024, p. 1). HJPs pursue objectives on various levels, including individual health outcomes, civil society reform toward greater accessibility for socially disadvantaged populations, and macro-level advocacy for more just societies. HJPs occur in different ways, including co-located legal services in primary and tertiary health care settings (Beck et al., 2022; Burrows et al., 2011; Moffatt et al., 2004; Woodhead et al., 2017), through referrals (Close et al., 2021; Ollerenshaw & Camilleri, 2017) and/or legal outreach services integrated with social care (Forell & Gray, 2009; Frost-Gaskin et al., 2003). Some models target specific subsets of the general population who are made marginalized in mainstream health and social care provision, including people seeking to access housing (Hernández, 2016), young people in regional settings (Ollerenshaw & Camilleri, 2017), people in palliative or end of life care (Close et al., 2021; Rodabaugh et al., 2010), or migrants and people from culturally and linguistically diverse (CALD)^1^ communities (Fuller et al., 2020; Moffatt & Mackintosh, 2009).

Specifically, people from culturally and racially marginalized (CARM) communities living in Western, White normative, and/or settler colonial societies experience structural and cultural barriers to psychosocial support aiming to improve their mental health (Costa et al., in press). Concurrently, they often confront many legal issues that may predate the experience of ill-health or emerge as a nearly inevitable result of social exclusion. Some such legal troubles are specific to people from CARM communities who often have a migration background. For example, people may have ongoing commitments toward their families and communities overseas (Singh et al., 2010) or confront uncertainty regarding migration and settlement status (Zanchetta et al., 2021). Other legal issues reflect broader trends in the general population, including overwhelming consumer debts (Gabbay et al., 2017), domestic and family violence (DFV) (Maturi & Munro, 2023), obligations in the local community (e.g., council fines), or difficulties accessing welfare entitlements (Greasley & Small, 2005; Sherratt et al., 2000). Yet, for people from CARM backgrounds, navigating legal, welfare, and health systems can be challenging due to a lack of cultural responsiveness in mainstream services (Balram et al., 2024; Mescouto et al., 2024). The culture—or way of categorizing and making meaning based in traditions and disciplines (Dune et al., 2021)—of mainstream health and social care services is often at odds with CARM communities. Culturally responsive care (and other services), in contrast, is sensitive to “the attitudes, feelings or circumstances of groups of people that share a common and distinctive racial, national, religious, linguistic or cultural heritage” (Dune et al., 2021, p. 288). Unresolved legal issues while facing social and political marginalization can exacerbate existing mental ill-health; in turn, poor health presents a formidable barrier to resolving legal problems and preventing their escalation (Pleasence et al., 2008).

The Australian Bureau of Statistics (ABS, 2023) estimated on 30 June 2023 that out of 26.6 million residents, 8.2 million were born overseas. In other words, just under one-third of people residing in Australia are first generation migrants, with many more citing at least one parent born overseas. Yet, their experiences with seeking culturally responsive care are not well understood. In this context, we explore how legal support is delivered through an HJP embedded in a culturally responsive mental health service operated by a non-government organization catering to people from CARM communities in Southeast Queensland, Australia (Olson et al., 2023). First, we aim to demonstrate the intersecting challenges experienced by people from CARM backgrounds at the nexus of legal, health, and social care, why culturally responsive services are needed, and the opportunities and challenges to their implementation. Second, we aim to illustrate how culturally responsive research practice can produce knowledge for advocacy, sustainability, and improvement of HJPs for CARM populations.

We begin by reviewing the evidence on HJP outcomes for individuals, communities, and health and social care systems. We then introduce the underpinnings of HJPs in fundamental cause theory (Cockerham, 2021) and draw on the notion of intersectionality (Crenshaw, 1989) to conceptually enrich our analysis. Then, we apply this theoretical lens to data produced through culturally responsive qualitative research for the purpose of evaluating the psychosocial support program. We comment on the potential pitfalls of connecting health with justice where current and historical elements of law contribute to marginalizing processes. We conclude with a discussion of the implications for culturally responsive research and care and raise questions to feed into the research agenda on HJPs.

### Toward Integrating Legal Services Into Health and Social Care to Address Social Determinants

HJPs are rooted in the public health movement addressing the social determinants of health. Reviews on the impact of these collaborations (Beardon et al., 2021; Martinez et al., 2017) found the emergent evidence base supports claims that HJPs are effective in identifying and resolving individuals’ legal issues, in turn addressing the underlying social drivers of poor health. Notably, Beardon and colleagues (2021) identified strong evidence for better mental health outcomes after legal interventions. Rigorous evidence on other HJP objectives, such as advocacy for systemic change, greater health equity, and better health services utilization on the population level, was less conclusive. A rapid review on the economic impact of HJPs (Granger et al., 2022) found that while financial returns for individuals supported with legal interventions were clearly documented, the cost-effectiveness and financial impacts of such interventions more broadly or how they affected service use were not consistently reported on.

Further engaging with the current scholarship on HJPs, we found little reflection on the historical and contemporary contributions of intertwined social, health, and law systems to marginalizing processes in settler and (post)colonial societies such as Australia (see Staines, 2023). This is an important area for study, given that individual-level legal support needs of people who are culturally and racially marginalized to some extent arise from system-level processes that constrain opportunities for good health, for example, in access to “universal” health care tied to migration status, or conditionality and exclusion from welfare entitlements.

There is also great variety in how HJPs are implemented. Legal services may be co-located, sought out via dedicated referral pathways, or integrated into multidisciplinary teams (Beardon et al., 2021). Martinez and colleagues (2017) noted that theoretical underpinnings of service development, implementation, and evaluation are often not made explicit. Moreover, even though legal services are in some fashion tagged onto health care, many such interventions do not explicitly do so under the label HJP (Granger et al., 2022). How HJPs can be configured to be culturally responsive is even less clear. This has implications for how intersections between legal and medical care are understood and what kind of knowledge comes to bear within interdisciplinary practice. Below, we describe the conceptual approach we have chosen for the study of integrated services catering to CARM.

### Interrogating Culturally Responsive Health, Social, and Legal Care Through Intersectionality

The most prevalent conceptual framework in the scholarship on HJPs draws on the social determinants of health literature (Genn, 2019; McCabe & Kinney, 2010). Popularized by Michael Marmot’s call to investigate and address the “causes of the causes” for social gradients in health (2005, p. 1101), this approach is anchored in fundamental cause theory (Cockerham, 2021; Link & Phelan, 1995; Phelan & Link, 2013). Fundamental cause theory queries why and how social structures promote good health for some, but not for others. Social categories are considered a fundamental cause for health outcomes under four conditions: First, the social category influences the experiences of multiple diseases. Second, the social category is exposed to diverse risks and pathways impacting disease outcomes. Third, the social category is characterized by differential opportunities to mitigate such risks through available resources. Fourth, the relationship of the social category with health outcomes is persistent, even though there might be advances in biomedical knowledge practices (Cockerham, 2021). CARM as a social category fundamentally affects individual and community health, and hence meets the criteria to be considered within the social determinants of health framework.

We add the notion of intersectionality to approaching CARM as a social category that constitutes a fundamental cause to trouble the “straightforwardness” with which HJPs address structural determinants of health. Continuing the philosophical trajectory of Black Feminist thought, Crenshaw (1989, 1991) distinguished three interrelated facets of intersectionality: structural, political, and representational (Carastathis, 2014). Structural intersectionality captures how different social categories of exclusion in their simultaneity have the capacity to cause harm to a person or community. Political intersectionality attends to the consequences of denying this simultaneity resulting in seemingly contradictory and divisive agendas. Representational intersectionality is concerned with the social reproduction of difference through harmful tropes. Carastathis (2014) notes, “intersectionality insists that multiple, co-constituting analytic categories are operative and equally salient in constructing institutionalized practices and lived experiences” (p. 307). In this way, intersectionality contains the principles of simultaneity, complexity, irreducibility, and inclusivity. Accounting for only one form of oppression (e.g., based on gender) neglects the enmeshed nature of how marginalization manifests for the person who is at the center of multiple, interlocking normative grids.

Transferring the concept of intersectionality to the study of support for CARM populations requires deliberation and reflection. For instance, the acronym CARM itself seems to signal a multiplicity of categories drawn upon to explain oppression. A successor to the much-critiqued umbrella term culturally and linguistically diverse (CALD) (Lakin & Kane, 2023; Maturi & Munro, 2023), CARM makes explicit the weaponization of cultural and racialized subjectification in the perpetuation of social exclusion. At the same time, culture and race are themselves categories containing vastly different sets of experiences and can be drawn on for positive identification recognizing shared strengths and belonging.

Accordingly, we take a careful approach to applying intersectionality, by tracing how legal, health, and social care systems become entangled in the provision of psychosocial support for CARM populations. We explicitly acknowledge that these systems harbor multiple capacities; they have the capacity to extend relations of care at the same time as they might capture people in relations of oppression. This dual capacity of macro-level social forces affects the opportunities for people at their intersections to address mental ill-health. With this tension in mind, we aim to produce knowledge on the specific legal issues people from CARM backgrounds face, how HJPs provide support to address these issues, and the challenges and limitations HJPs and their service users encounter in practice.

## Methods and Materials

Our study poses the following research questions:

RQ1: What legal issues impact the health of people from CARM communities?
RQ2: How are interrelated legal and health issues addressed within the HJP?
RQ3: What principles underpin HJP practice for people from CARM communities?

To address the research questions, we explore the perspectives of service providers and service users of a psychosocial support program that attends to legal issues through a culturally responsive HJP. We refer to this program as “Mosaic Care” to preserve anonymity.

### Study Design, Setting, Recruitment, and Data Collection

The data on which we base our analysis were produced in the context of a program evaluation of specialist psychosocial mental health support reviewed and approved by The University of Queensland’s Human Research Ethics Committee (HREC ID 2022/HE001102). Funded by the state government, Mosaic Care’s overarching objective is to improve the social, emotional, mental, and physical well-being of people from CALD [sic] backgrounds in Queensland, Australia. To that end, it provides multifaceted supports understanding mental health as embedded across and within multiple systems. Through Mosaic Care, service users can access welfare and legal assistance by leveraging an HJP with a local community legal center, or through referral pathways to specialist welfare, housing, employment, or DFV services. Thus, supports available to service users are not limited to counselling, behavioral, and wellness interventions, but also address social drivers of mental ill-health. Mosaic Care seeks to be culturally responsive by offering to match service users and service providers appropriately to accommodate service users’ needs and preferences. Services are provided primarily by staff with migration experience, including humanitarian migrants and asylum seekers. Multicultural peer support workers, who are often trained health and/or social work professionals, support nearly all program activities. However, providing services via staff with shared experiences, culture, and language is only one component of culturally responsive care. We identify Mosaic Care’s program configuration along service principles, practice frameworks, and values of culturally responsive care in the Results section.

We closely collaborated with key staff members at the organization running Mosaic Care to progress the research in a culturally responsive way. We took time to engage deeply with the history and vision of the organization running Mosaic Care and the organizational cultures in which the HJP is embedded. We were guided by their knowledge and expertise gained through their relationships with the people they support in identifying and approaching prospective participants for recruitment. Prospective service provider participants and partner organizations were identified via a mapping exercise during a half-day workshop with the evaluated organization. During this workshop, we also developed a strategy to recruit service users employing a purposive sampling strategy (Daniel, 2012). We aimed to arrive at a sample of diverse experiences with Mosaic Care (i.e., in terms of length and types of supports) and demographic characteristics (e.g., legal migration status, country of origin, and language). The final sample included 25 service providers affiliated with the evaluated organization (internal), 12 service providers partnering with the evaluated organization (external), and 16 service users.

All participants provided written informed consent prior to enrolment in the study. We undertook 39 semi-structured interviews employing three separate interview guides for these participant groups to explore experiences of delivering services, partnering with and receiving support from Mosaic Care (see Appendices A–C). Interviews took place between August 2022 and November 2022 in person, over telephone, or via videoconference depending on participant preferences. Likewise, to accommodate organizational and individual schedules and priorities, we used a mix of group and individual interviews (Table 1). Interviews lasted between 16 and 83 minutes (average 45 minutes). We audio recorded all interviews with permission from participants. All participants received a $30 to recognize their contribution to the research.

**Table 1.** Data Collection and Sample.

*n* = 53	Service Providers (Internal)		Service Providers (External)		Service Users	
Individual interviews	6	*n* = 6	8	*n* = 8	14	*n* = 14
Group interviews	6	*n* = 19	2	*n* = 4	1	*n* = 2
All interviews	12	*n* = 25	12	*n* = 12	15	*n* = 16

### Reflexivity in Culturally Responsive Qualitative Research

As authors, we represent diverse cultural ties and nationalities. As academics affiliated with a large higher education institution, most of us have migration backgrounds. Some hold more than one citizenship, have firsthand experience of the complexity of Australia’s health system as migrants and/or service providers, and live and work as non-native speakers in an English-speaking country. We recognize how our experiences are marked by the privilege afforded by our roles as academic researchers and migrant settlers on unceded Indigenous lands. From this position, we appreciate the heterogeneity among people and communities subsumed under terms such as CALD and CARM. Working on this project, some of us were reminded of our feelings of being categorized in acronyms such as CALD or CARM when settling into Australian society—a taster of being othered that we were mostly unaccustomed to in our countries of origin.

We also represent diversity in terms of disciplinary backgrounds, including sociology, social work, and physiotherapy. We bring these varied experiences to our analysis, seeing this as a strength within our culturally responsive qualitative research (Jordan & Hall, 2023)—research that aims to foreground cultural dimensions in research design and knowledge production processes—as we are engaged in ongoing dialogue, stretching the epistemological and ontological boundaries of our respective disciplines. The varied trajectories to this research also motivate our intentions to work toward greater health equity by fixing systems that people must necessarily rely on to live and thrive, and yet often cause harm to people by the way they are designed. We sought to maintain culturally responsive qualitative research practice at every stage of the study, including data collection. We gave all prospective participants the option to facilitate interviews in their preferred language irrespective of their English language proficiency. This resulted in eight service user interviews relying on a telephone-based professional interpreting service. A further three service user interviews relied on the evaluated organization’s peer support workers to facilitate the interview in a language other than English. These included the following languages: Spanish, Turkish, Vietnamese, Arabic, Indonesian, Amharic, Farsi, and Tamil.

Reflection on our research practice sensitized us to issues that might unfold in health and social care. For example, often there was little time to brief interpreters regarding the relational nature of a research interview. At times, long participant answers were condensed into a concise response. In turn, prompts and follow-up questions asked by the interviewer to delve into greater depth were sometimes translated to simply repeat the original question. We also observed that while participants preferred to express their views in their first language, they were often capable users of English, and some shared their frustration with interpreters after the interview. Interpreters also articulated frustration with interview settings, for example, the presence of young children, friends, and family audible over the phone line. On one occasion, a member of the research team conducted an interview in Spanish due to technical difficulties with phone interpretation. Conducting an interview in a language that while fluent was unfamiliar to the interviewer deepened reflections on the role of language in culturally responsive research practice. While errors of expression were a source of embarrassment for the interviewer, the participant responded with patience and support. We interpret this as accidental opportunity for reciprocity in the research, highlighting the relational nature of knowledge production in which language is just one of many factors.

To stay true to our intention to conduct the research in a culturally responsive fashion, we persisted in allowing for the messiness of everyday life to be reflected in these interviews. We had to accommodate participants’ caring commitments in the research setting to prevent their exclusion, even if it meant that participants were also attending to their children while sharing their insights. Often service users were more concerned about feeling at ease than about privacy and invited trusted others, including peer support workers to be present. Rather than a challenge to the rigor of the evaluation as we had feared, we found this to be an invaluable asset which provided us the opportunity to observe the relationship between service providers and service users during interviews, while also enhancing a sense of safety for these participants during the research process.

### Data Analysis

The interviews were professionally transcribed and deidentified for analysis. To make sense of the data, we employed theory-driven (i.e., deductive) thematic analysis (Braun & Clarke, 2020) informed by our operationalizations of the social determinants of health and intersectionality frameworks and following the quality criteria specific to thematic analysis (Braun & Clarke, 2021). Our approach falls within reflexive thematic analysis, as we developed themes from codes relying heavily on the interpretative work of the research team in dialogue with key stakeholders affiliated with the organization operating Mosaic Care (Braun & Clarke, 2020; National Health and Medical Research Council, 2018). We paid close attention to the subjective position of us as researchers and our active role in constructing codes to arrive at themes. We make explicit the theoretical assumptions on which the analyses are based, in particular how we position CARM as social category within fundamental cause theory.

In two half-day workshops with stakeholders, we presented tentative findings, gathered their perspectives that further enriched or prompted revisions to the evaluation report, and provided opportunities to reflect on implications for practice and policy. Subsequently, these findings have also been reported elsewhere, including a number of these stakeholders as co-authors (see Costa et al., in press; Mescouto et al., 2024). The present article continues this work taking a different analytical avenue.

The role of legal support as part of psychosocial care toward improved mental health for people from CARM communities was not initially of interest to the study. However, as the data analysis progressed, the salience of addressing legal issues at the intersection of health and social care became apparent, particularly given the active HJP involved in service delivery at Mosaic Care. While the first author led the analysis through the stages of familiarization, coding, and initial theme development, the research team iteratively contributed to discussion, further development, and refinement of the themes. Consequently, this subtheme was elevated to a key analytical focus prompting us to revisit the dataset as a whole through the conceptual lens of intersectionality (Crenshaw, 1989) complementing the social determinants of health perspective. In the section that follows, we present findings on the role of legal support in delivering psychosocial services to people from CARM communities as part of addressing their mental health needs. All quotes are deidentified using pseudonyms, and indicate the role of the participant as either internal service provider, external service provider, or service user, and whether they are from individual (“I”) or group interviews (“G”). We considered cultural appropriateness during our process of assigning pseudonyms, choosing pseudonyms that were culturally similar to the participant’s real name but sufficiently different for anonymity.

## Results

First, we describe the variety of legal issues service providers and service users encounter and how they affect opportunities for good health. Second, we provide insights into how care coordination with practitioners providing legal support takes place. Third, we identify the service principles and values that inform these practices. Throughout, we tie these insights together within the intersectionality framework, in particular with respect to its structural, political, and representational facets.

### Structural Intersectionality: Legal Troubles? Health Troubles? Or Both?

We sought to gain an understanding of the most pressing concerns that service users and service providers encountered during service delivery. We asked service providers what support needs they addressed commonly, and likewise we prompted service users to tell us what they wanted support for. Even though not explicitly phrased in terms of legal needs, responses to our questions revealed a gamut of legal issues entwined with mental and physical health. This service provider shared:

Immigration matters. That might really change everything for them, especially if they have a partner that’s going to be deported soon and they’re not going to have that partner there. (Lucia, internal service provider, G)

This service provider also discussed how migration and settlement not only affected opportunities for family unity but also impacted how families navigated domestic violence (DV) and separation:

A lot of our client is a combination of DV support that they need and often DV related to family law. … sometime it’s housing is another one, financial support, relationship conflict that’s impacting families and client wellbeing. … our client also got a lot of issues with immigration, because sometimes they come as refugees or they got married to someone here and then the relationship ends. (Margo, internal service provider, G)

The precarity of settlement status highlights the additional and intersecting forms of marginalization. We see at play here, structural intersectionality, in which belonging to the social category of “migrant” or “refugee” coincides with other social categories such as a “person experiencing financial hardship or domestic violence” compounding the potential for harm to a person. Being a person whose presence in Australia is conditional, for example, on being in a relationship with a primary visa holder, permanent resident, or citizen, or being financially secure was complicated by the entanglement of health with legal issues:

I was also worried about my visa and [support worker] said to me, “… the government’s not going to get rid of you tomorrow just because your ex wants to cancel your visa.” …. I didn’t know what to do, I didn’t know anything about the legal side of things or the emotional side of things, I didn’t know how to ask for help or how to deal with the psychologist. (Manuel, service user, I)

Participants, like Manuel, articulated a heightened sense of intersecting vulnerabilities after separating from a partner. While financial constraints, loss of income, and housing insecurity as the result of separation are common for many people, especially women (Kuskoff, Parsell, Stambe, et al., 2024), these participants confronted the prospect of having to navigate systems they felt ill-equipped for:

I never been educated, not in my language and not in English. I’ve been here since mid, early ’80 and I learn English through my cleaning job. (Eva, service user, I)

I had some mental and emotional health problems because of my financial difficulties. … I had a problem with a lady and then [support worker] helped me to find a lawyer to just settle the problem with that lady, … I have lots of debts. … I have done citizenship test twice, and I couldn’t pass. … If I’m not in the pass [next time], bye-bye. (Azar, service user, I)

My workplace, they terminate for me because they have bullying, harassment. … they’re treating me very badly. … at one time I tried to suicide … I’m thinking badly and then I don’t know where I go, what I do. I don’t know because I don’t have a money to find a legal way or whatever. That why I’m fairly upset. And then I’m still refugee. (Thani, service user, I)

Structural intersectionality among participants eventuated as being put at risk of being taken advantage of and the fear of having to leave behind the country they had come to call home. What is more, participants explicitly connected these legal issues with their experiences of ill-health, revealing additional burdens people from CARM communities need to resolve. Coping with a toxic workplace is difficult enough, yet even harder when there is no legal remedy at one’s disposal. In Australia, a recent report found that it is virtually impossible for migrant workers to hold their employers accountable to contractual obligations, including basic entitlements to pay (Hemingway et al., 2024). Taking legal action against workplace bullying carries costs and risks that can jeopardize one’s future opportunities to reside in Australia.

We also note the financial burden participants consistently talked about across the sample. Australia is currently experiencing unprecedented housing and cost of living crises that particularly impact people who are already socially and economically disadvantaged (Plage et al., 2024). However, people from CARM backgrounds are doubly affected by these crises as their entitlements and access to welfare supports are comparatively constrained. This includes access to the tax-funded universal health care system, Medicare, and benefits for unemployment and disability. Even people who are eligible to these entitlements in alignment with their settlement status might still miss out because of overly convoluted application processes (Balram et al., 2024). Seeking welfare advice, legal representation, or resolutions taking judicial pathways requires savviness, resources, and patience, which may deter people from CARM communities disproportionately, exacerbating their ill-health. Many service users and service providers recounted how free legal support made available through Mosaic Care not only helped with navigating the complexities of rights and obligations but also significantly improved their health:

[They] assisted with some of understanding the court processes and how it all works and basically gave me some reassurance. … I was in a depressive and isolation mode where I had no one, nothing, and these ladies actually gave me a very good insight in what I needed to do. … It was a long and stressful process, especially the court proceeding [for my children from the separation]. (Elif, service user, I)

When I’m manic I couldn’t control financially, which is one of my bipolar symptoms. … my debt’s there. So once it’s all cleared out, I felt very emotionally light … My short-term goal is more getting healthier mentally and physically. And my midterm goal is finding a place to live through the government housing, as well as finding a job. … at least I have a hope now. Just couple of months before I had no hope, so hopeless and so worthless. You know? (Bridget, service user, I)

The quotes above reveal the diversity of legal issues faced by people from CARM backgrounds and how these issues affect their health. While things like migration and settlement are distinct to their experiences, troubles with housing, employment, and family are more universal. However, as we have shown, while commonplace, intersecting trajectories of marginalization are not reducible to being either a migrant, a disenfranchised worker, or a person with mental ill-health or disability but can only be addressed comprehensively. Below we elaborate on the integration of legal support into health and social care in this context.

### Political Intersectionality: Health and Social Care Coordination for CARM Populations Involving Health Justice Partnerships

Political intersectionality conceptually deals with the consequences of downplaying or denying the synchronicity of compounding socially disadvantaged categorizations. Intersectionality needs to be addressed in care coordination across multiple service providers located in different organizations across government and non-government sectors, as this service provider observed:

Wherever you go, there’s a visa restriction .… people are on this kind of merry-go-round of in and out of the system. That’s not serving them. And that’s not unique to people from CALD backgrounds, but it has an extra layer of complexity because of the lack of cultural understanding … there’s a lot of health injustice that people experience on their pathways .… whether it’s in employment, whether it’s discrimination, whether it’s oversees qualification, lack of recognition. (Terrie, internal service provider, G)

Low-lighting the complexity and interrelated nature of these issues undermines the capacity to address them comprehensively. Being interdisciplinary and cross-sectoral, how HJPs are enacted can have significant flow on effects for experiences with services and service user outcomes. There are various models according to which legal support features within health and social care. While some are implemented through flexible (and somewhat ephemeral) referral pathways, other HJPs integrate paralegals or lawyers firmly in a co-located model of primary health care (Beardon et al., 2021). Either approach is centered on primary health care as the focal point for any type of support, as this service provider reasoned:

Primary healthcare is just such an *entry point* for the multicultural population, not just for mental health .… [Mosaic Care] transitioned into a psychosocial support *front door* …. this nexus between people’s sense of health and wellbeing in terms of their mental health, and other social determinant-related issues … workplace injuries, people not knowing their workplace rights, being on temporary visas and how that’s all playing on people’s physical and mental health, and the need for an *entry line* like this being so important to at least *hold* people while they’re trying to work through those issues in the absence of culturally aware and responsive services in their locality. (Terrie, internal service provider, G—italics added for emphasis)

In this service provider account, primary health care is represented as “entry point,” “front door,” or “entry line” for issues that go beyond the remit of biomedical approaches to health. These are explicitly located within the social determinants of health framework that alludes to the intersectional nature of different forms of marginalization. We note a “holding” approach expressed here that mitigates the potential impact of political intersectionality. Medical treatments are administered to alleviate suffering while the drivers of this suffering are identified and worked on. As this service provider explained, challenges occur when intertwined legal and health issues are separated out:

Look at our health justice stuff, right? So a person who’s got a chronic health condition and is experiencing DV or something like that, you’ve got your health professionals going, “Well, that’s a legal problem,” and then you’ve got the lawyers saying, “Well, that’s a health issue.” (Charlie, internal service provider, G)

There is the potential that the dual and intersecting nature of issues results in not addressing either of them. Being aware that the clinical service cannot resolve the “causes of the causes” that drive poor mental and physical health (e.g., DV, settlement, or housing), clinical service providers sought within their scope of practice to offer targeted support without minimizing the interlinked nature of law and health. This placed them in a difficult position where they had to manage service user expectations, while sustaining relationships of trust with them. For instance, one of the clinical service providers shared how a service user came to believe she had clout with immigration officials after initiating a phone call with them on the service user’s behalf. Balancing these expectations with the realities of government procedure was a common challenge for care coordination that within the service team was tackled by calling on multicultural peer support workers and specialist services. These service users noted:

I find it very difficult to submit things from the hospital concerning our status here in Australia … [support worker] was the one who tries to help us in our papers, in submitting to the immigrations and telling them of the problem that we went through and faced. Yeah, and she was there. And she passed us to their legal advisor, who’s been helping us till now. It’s a blessing to us. (Dewil, service user, I)

I have an intellectual disability. And you would appreciate that intellectual disability has a very impact on my life and the way I process things. Where, in this timeframe, [support worker] did an exceptional job of understanding me and taking me to the right services, like NDIS. (Elif, service user, I)

On that first call I had a panic attack and didn’t know what to do with her and I felt like I was in a hole, there was nothing beneath me, and I felt all alone. And [support worker] told me many times that I’m not alone. … [she] also found for me that phone number [DV hotline], for me to call, for them to then guide me. (Manuel, service user, I)

This team-based approach internal to the organization and linked in with external health justice partners offers some relief for clinical primary health care staff, while also providing support to service users beyond clinical care. Given the intersecting forms of marginalization faced by many of the service users, this was crucial to providing care capable of improving mental and physical health. This approach addressed the psychosocial issues causing feelings of extreme isolation, confusion, or even panic, that exceed the remit of health professionals, as this clinical service provider explained:

Patients who have come either by boat or also by plane … from offshore community detention and onshore community detention, released into their community with little to no financial or social support … [They need] medication and payment assistance, food, food assistance, radiology/pathology payment assistance, yeah, and also linking in with trying to find them housing and sometimes case management … our colleagues … work with us closely and we try and identify that gap while we are serving them from a mental health perspective. (Maxi, internal service provider, G)

A legal service provider partnering with Mosaic Care clarified:

We’re a small team who assists vulnerable clients to navigate through … legal processes. … [Mosaic Care] may contact us seeking legal assistance for one of their clients and that’s generally how we receive referrals … I’ve also referred a client to the service for counselling supports. … it’s an informal partnership. (Jane, external service provider, I)

Importantly, we locate these practices of integrating legal support, access to legal representation, welfare advice, and logistical support within practices of culturally responsive care (Dune et al., 2021). The multipronged approach ensured that service users were not simply put *on hold* but *were held* while working toward addressing the psychosocial drivers of their health problems. Service users’ distress was often acute at point of contact with the services. Rather than applying a demedicalized understanding of social issues to focus exclusively on the social determinants of ill-health (e.g., migration, DV, finance, and welfare), we demonstrate that indeed clinical and non-clinical services working across paradigms were needed to respond comprehensively to service users’ needs. Below we explore the principles underpinning this integration of services to provide culturally responsive care.

### Representational Intersectionality: Principles for Culturally Responsive Care

Intersectionality is also relevant for how people from CARM communities are represented, for example, through the systemic reproduction of harmful tropes in culture and advocacy. So far, we have primarily applied the conceptual tools of structural and political intersectionality in how Mosaic Care pursued individual health and justice outcomes through care coordination with internal and external specialist services. Here, we tease out underlying principles that inform care coordination and cut across different societal impact levels. Advocating on behalf of service users for system transformation was considered core business. These service providers explained:

That non-clinical aspect of [our team] is core to what we do. So, we’re not having structured, like [our other team], that kind of more psychological, psychotherapies, CBT stuff, but we’re very much non-clinical, looking at the whole person to de-stigmatize what our culture puts onto CALD communities as mental ill health. (Charlie, internal service provider, G)

This service provider explicitly addressed representational issues, in situating their work within concerted efforts at de-stigmatization. Mental ill-health is carrying stigma in Western and CARM communities; however, stigma and its consequences materialize differently across socio-cultural contexts. What is assigned the quality of an individual pathology in universalist mental health parlance can be made sense of as a collective phenomenon through a cultural relativist lens (Brossard & Chandler, 2022). Consequently, language and culture became the arena in which representational intersectionality took center stage, as in the following example:

We become aware that the lawyer was not even using the interpreter much. They were texting to each other, emailing, and the person doesn’t speak English, so she has to translate on her phone …. if we got our lawyers or whatever, then it much easier. You book the appointment, the client come back, explain everything, and then they get ready for the court … we also have our interpreters on a phone we can use on call. And then we have extra [multicultural peer support workers] who provide a cultural and the language to support. (Margo, internal service provider, G)

This exemplifies how people from CARM communities can be disadvantaged in the legal system, even when they have access to legal representation. Not only does the integrated HJP consistently offer interpreters, it also brings peer support workers into the process in the explicit acknowledgement that language translation on its own does not constitute culturally responsive care or mitigate representational intersectionality. The below excerpt illustrates this point:

A couple were separated, but they didn’t understand the reason why they need to leave each other. Whatever’s happened … neighbours called the police, police came, handcuffed the man, took him away, left the lady, nobody provide any interpreter or any legal support, so they never knew they are entitled to go and to defend. Now they have a DVO [domestic violence order] for five years, and yet nobody explained to them of their right, their responsibilities. (Margo, service provider, G)

When it comes to legal issues with their distinct lexicon and logic, assumptions of universalism can have profound consequences. We do not claim to know what occurred to trigger the neighbor’s call to the police and neither do we subscribe to any justifications of DFV within cultural frames of reference (Maturi & Munro, 2023). In turn, outcomes of bystander intervention are highly context dependent (Kuskoff & Parsell, 2024). What we note in this account is the absence of culturally responsive follow-up care, including support to the couple that would empower them to understand their legal rights or how their experiences were framed and addressed within legal discourse.

Employing interpreter services in combination with peer support workers, hence, is a key principle of service delivery that needs to inform practices of care to mitigate the harms and risks of intersecting marginalizations. Mosaic Care actively encouraged their partners to adopt such approaches in a strategic initiative toward system improvement, yet always centered the whole person they are supporting. These service users shared:

It’s holistic to me, because it touches my health, it touches the legal part of our stay here, and it also touches our family as a whole, because it helps my son, and it also meet my physical needs because it organized food from [charity] to help me. And also, they organize a place for me to stay. (Dewil, service user, I)

Each person is different. My circumstances were I was having a baby and it was financial, but other people will have other issues. … [Mosaic Care] follow ethical rules and they’re responsible and that they follow up and they’re able to provide assistance depending on the situation that you find yourself. (Marta, service user, I)

These service users felt understood and supported across aspects of their lives that went well beyond the clinical remit, and addressed the challenges they encountered in their everyday lives. To avoid the pitfalls of misrepresentation alongside meeting their practical needs, the integration of legal support into relations of health and social care was instrumental. However, relations of care need time to be nurtured (Plage et al., 2023). This service user reflected:

Sometimes I’m not answering [support worker’s] phone, which is very rude, but I really, really didn’t want to talk to anyone at that time. Then [support worker] leaving the voice message or text message, like it’s okay, she can understand, and some encouraging words and rescheduling …. they fully understood my sickness .… they’re very friendly, they’re very caring, and they really giving attention on my own interest …. I feel like, oh, someone got my back. (Bridget, service user, I)

Support here focused on individual need and served to avoid tropes, such as those linking deservingness with gratitude that hold behaviors of people with migration backgrounds to a higher moral standard (Clark et al., 2023). In addition to the key principles of centering on their personal support needs to provide trauma-informed care and drawing on the cultural expertise of peer support workers, we also found that service providers sought to practice a strength-based approach in the social work tradition (Brun & Rapp, 2001):

Something a client can do on their own, we’ll let them, and we watch them do it. And other things they need support with, or they need link with other service providers to help them manage those things. So, we will tell them where to go, sometimes we just link them ourself, and we will advocate for them …. it’s not about us just working with them, it’s about them working with us. … They’re just not part of the problem but they’re also part of the solution. (Lucia, internal service provider, G)

The philosophy of [Mosaic Care] is about, we don’t turn people back to say, “No, we don’t.” We just try and help in another way. It’s a duty now, as we’re trying to do our best. (Charlie, internal service provider, G)

These service providers articulated a strong commitment to supporting service users through a low-threshold, accessible service model. This was partially grounded in their understanding that many of the service users had few other options to find support that would take intersecting marginalization into account and offer culturally responsive care that would also address the impact of injustice on their health. However, while this approach to care steered clear of deficit-heavy tropes of people from CARM communities as dependent and vulnerable, this is often hard to action within the convoluted, fragmented, and programmatic nature of the larger health and social care systems:

I tried to explain to [service user], “We are not the system. We are basically advocating on your behalf. Our role is this.” He was so caught up, system-dependent, the [police], the housing, horrific injuries. We understand that, but I had to make a clear decision, “We cannot work with you. We won’t help you. We cannot. Especially if you consider us to be the system that’s damaging you, we don’t want to damage you more than what you’re gone through.” And he agreed. … doesn’t happen quite often, but it does happen. (Maxi, internal service provider, G)

Service providers themselves are embedded into this landscape as they attempt to work with service users and advocate on their behalf. They confront limitations in their capacity to support service users when their previous experiences with government systems have irrevocably—and understandably—eroded their trust in health and social care. While principles of person-centered care acknowledged the often-traumatic experiences that service users had in their lives and how they affected their capacity to engage in care, we see here the dual capacity of interlinked systems to occasion culturally responsive care *and* to further cultural and racial oppression. Operating within system constraints sits in tension with the larger HJP vision to transform health and social care toward greater equity or may even work to inadvertently (re)produce some of these injustices.

## Discussion

Better integration of health and social care is increasingly recognized as crucial to the promotion of greater health equity, yet its potential to enhance culturally responsive care—care that is sensitive to and centers the needs of diverse cultural groups—is not well understood. HJPs are proposed as a way forward to attend to legal needs within clinical settings and beyond. While available evidence on the positive impact of HJPs for people experiencing social exclusion is encouraging, such emerging interprofessional and multidisciplinary models of “doing health” alongside “doing justice” call for culturally responsive qualitative research to produce knowledge on the opportunities and challenges for culturally and racially marginalized people. We complemented the dominant approach to HJPs within the social determinants of health framework to experiment with theoretical offerings from applying an intersectionality lens operationalized in structural, political, and representational intersectionality. Drawing on data produced as part of the evaluation of a culturally responsive psychosocial support service for people from CARM communities in Southeast Queensland, including interviews with service users, service providers, and partners external to the program, we found that the legal troubles faced by service users are similar to those found in other cohorts but complicated by cultural and racial marginalization. We identified how the complexity of navigating discrete yet interlinked health, legal, and social care systems is likewise further compounded if cultural and racial marginalization is not explicitly recognized in its impact or downplayed and minimized. We also described the service principles and values of culturally responsiveness in Mosaic Care and the embedded HJP, and how they seek to anticipate and reduce the risk of perpetuating harmful ways to represent people from CARM communities. We discuss findings in terms of opportunities, limitations, and challenges to advance justice in health through culturally responsive qualitative research.

First, we situate our findings and their interpretations vis-à-vis the extant scholarship on HJPs. Genn (2019) notes the diversity of legal issues that are encountered by marginalized people on the one hand and the range of models with which HJPs are practised to address them on the other hand. Legal issues range from financial difficulties (including debts), welfare access, family breakdown (including DFV), housing (including access and condition), employment, migration and settlement, and criminal justice contact (see Frost-Gaskin et al., 2003; Gabbay et al., 2017; Greasley & Small, 2005; Moffatt & Mackintosh, 2009; Moffatt et al., 2004). While there is often overlap between these legal problems in the lives of people experiencing social disadvantage, nonetheless varying vectors of marginalization result in distinct sets of challenges. CARM populations are a case in point, as their opportunities for good health and legal resolution face further complexities (see Hemingway et al., 2024). Not only do they additionally need to contend with migration and settlement law, but they also face the challenge of navigating health, legal, and social care systems that are configured along White and European values and norms (Brossard & Chandler, 2022; Mescouto et al., 2024). Applying an intersectionality lens, marginalization along racial, ethnic, and cultural logics can be seen to compound with social exclusion based in classed and gendered categories (Carastathis, 2014; Maturi & Munro, 2023). Such intersectional positioning requires targeted and nuanced HJP responses capable of promoting greater health equity for people from CARM communities by removing and mitigating obstacles such as linguistic and cultural discrimination, system complexity, and producing culturally responsive health and social care services integrating legal support. Mistrust of services is a challenge for many from CARM communities, especially those on humanitarian visas (Barudy, 1989); empowering relationships are imperative to intersectionally improving health equity. The findings from this study suggest that limiting HJPs to establishing referral pathways to specialist services, for example, to address DFV, is more likely to succeed if service providers have established and trusting relationships and are willing and resourced to collaborate closely for often extended periods of time.

Culturally responsive service delivery for people from CARM communities needs to draw from multiple frameworks, cutting across strength-based, person-centered, and trauma-informed approaches to care (Olson et al., 2023). In this way, we argue for making sense of HJPs in terms of an extension of the care paradigm eventuating in legal support beyond the social determinants of health. We also argue for greater engagement with the role of social work in the implementation of HJPs. Most service provider participants in this study were neither trained paralegals or lawyers nor health care professionals, but qualified social workers or human services professionals, often working as multicultural peer support workers, who also brought in their own lived experiences to address the socio-legal troubles service users face. While it is beyond the scope of the present article to provide an in-depth analysis of the benefits and limitations of peer support within culturally responsive health and social care integration, we argue that culturally responsive care leveraging peer support work is a meaningful addition to the frameworks listed above to attend to the intersection of racial, classed, and gendered marginalization. Future research would do well to flesh out the contributions of peer social workers from CARM communities within specialist HJPs.

The latter is crucial, specifically with respect to the cultural frames of reference to make sense of what is to be considered a health or legal issue. For example, we heard claims from participants that victim-survivors of DFV sometimes did not self-identify as experiencing DFV (Maturi & Munro, 2023 presenting a critique). Interventionist approaches imposing a Western understanding of and response to DFV might put victim-survivors at risk of housing instability, ostracization from community, or having children removed from their families (Kuskoff, Parsell, Plage, et al., 2024). In turn, dismissing DFV when it occurs with reference to cultural idiosyncrasies expresses “an essentialist view of culture” that risks the health and lives of those who are subjected to it (Maturi & Munro, 2023, p. 151). It invites both relegation (i.e., downplaying importance) and delegation (i.e., to community leaders and peer support workers) of the problem of DFV. HJPs supporting culturally and marginalized individuals, families, and communities need to navigate such perceptions to address DFV in a culturally responsive manner.

Finally, our findings also raise questions regarding the HJPs’ capacity for systemic transformation given their embeddedness in the service landscape. Many of the issues the HJPs sought to resolve through legal support and advocacy on behalf of service users stem from fundamentally unjust legal, health, and social care systems in the first place. Consequently, while practicing in line with the principles outlined here and cutting across social work, health care, and social justice frameworks is hoped to lead to greater permeation of integrated legal, health, and social care across the government and non-government sectors, a much more radical transformation is needed to remove drivers of mental ill-health.

There are important lessons to be learnt from our experiences undertaking culturally responsive qualitative research on this study. First, we were constantly reminded of the homogenizing capacity of social categories such as CARM. Negotiating this required ongoing deliberation and reflexivity throughout all stages of the research process to identify commonalities in experiences and perspectives of individual participants without generalizing from them or drawing conclusions about whole communities. Second, we were acutely aware of the logistical challenges stemming from the demands of culturally responsive qualitative research. Without the sustained in-kind support to this research from countless support workers and leaders in our partner organization, this study would not have been feasible. Yet, this type of support is hardly catered for within academic research institutions, putting additional strain on already thinly stretched budgets and workers in the non-government sector. We conclude that to enhance culturally responsive qualitative research practice, structural changes in the research and higher education sectors are needed.

## Conclusion

Health and social care integration is a perennial challenge for care practitioners siloed by funding streams and time-limited-service delivery. HJPs as a practice-led movement bringing together different sectors, disciplines, and worldviews represent a point of departure to enhance capacities to address social determinants of health. This study interrogated the praxis of HJP work in the context of CARM people’s mental ill-health employing intersectionality as a conceptual tool to makes sense of this phenomenon. In this way, we contribute to the emerging evidence base on the impact of HJPs by teasing out nuances in their implementation. HJPs are predicated on lasting relations between service users and partners inflected by commitments to care and social justice. Configurations of HJPs need to account for the specific needs and preferences of the populations they serve. In the case of legal support for CARM, this needs to be guided by a combination of interdisciplinary frameworks including trauma-informed, strength-based, and person-centered approaches enabling culturally responsive care in which legal, peer support, and clinical staff need to be accommodated. Where such diverse practitioners collaborate as partners working toward greater health equity through individual, meso-level, and macro-level advocacy, HJPs represent an antidote to some effects of intersecting marginalizations.

## Supplemental Material

Supplemental Material - Justice in Health? Studying the Role of Legal Support in a Culturally Responsive Mental Health Service in Australia Supplemental Material for Justice in Health? Studying the Role of Legal Support in a Culturally Responsive Mental Health Service in Australia by Stefanie Plage, Rebecca Olson, Nathalia Costa, Karime Mescouto, Sameera Suleman, Asma Zulfiqar, Jen Setchell, and Rita Prasad-ildes in Qualitative Health Research

## Note

1 We acknowledge the critiques in regard to the “culturally and linguistically diverse” (CALD) terminology and express our preference for the more recent term “culturally and racially marginalized” (CARM). Throughout this article, we use CARM when we refer to our own research and CALD, if this was the heuristic used in the cited research or policy.
