# Supplementary material for: Justice in Health? Studying the Role of Legal Support in a Culturally Responsive Mental Health Service in Australia
Source: Qual Health Res. 2025 Apr 2;35(4-5):418–32. doi: 10.1177/10497323251315435 (PMC11967093; doi:10.1177/10497323251315435)
Supplement: Supplemental Material - Justice in Health? Studying the Role of Legal Support in a Culturally Responsive Mental Health Service in Australia [file sj-pdf-1-qhr-10.1177_10497323251315435.pdf]

Appendix A

Service User - Interview Guide

Today I would like to hear about your experience with [this] program, for example, how you got involved, what services you accessed, what you found most useful, and what could be improved.

1. To start, tell me about how you heard about *[Mosaic Care]*? PROBES: Was it easy for you to know these services existed? Did someone encourage you to look them up? Were you referred from hospital or another service provider? Did you hear about it through someone who used *[Mosaic Care]'s* services? Did you see it advertised somewhere?

2. How would you describe the purpose of *[Mosaic Care]* to a friend who had not heard of it before?

3. What *[Mosaic Care]* services have you used? PROBES: clinic? hotline?

4. Can you tell me a little bit about your reasons for accessing these services? Since when? Did you feel the services met your needs at the time? And now? PROBES: What do you hope to get out of these services? For yourself? For others? [interviewer instructions: gentle questioning to let participant guide the talk about their mental ill-health]

5. What do you do as a *[Mosaic Care]* client? PROBES: How do you access the services (e.g., in person, telehealth, both)? How often did/do you attend scheduled appointments? How helpful are these?

6. Are you currently accessing *[Mosaic Care]* services? PROBES: IF NO: Can you tell me why you are no longer using these services (e.g., no longer needed)? Do you plan on continuing with the services at a later time? What would you likely need to participate again? IF YES: Can you tell me what makes you come back to *[Mosaic Care]*?

7. Tell me what made/makes it difficult to access *[Mosaic Care]*? PROBES: Language options? Cultural appropriateness? Safety? Timing? Frequency? Costs? Transport? Mental/physical health? Commitments? Other? If they experienced difficulties, ask: How did you manage those?

8. Were/Are you also accessing other services to support your mental health other than *[Mosaic Care]*? If yes, tell me a little bit about these services. PROBES: How do *[Mosaic Care]* services complement or overlap with other services? What makes *[Mosaic Care]* different from others? How important is that for you?

9. What changes have you noticed in your life since using in *[Mosaic Care]'s* services? PROBES: Feeling supported? Stronger family relationships? Your wellbeing? Safety? Has/Is *[Mosaic Care]* had/having any impact on other people in your life? Would you recommend *[Mosaic Care]* to others?

10. What do you think *[Mosaic Care]* could do better to support you? Overall, in your experience, what would you say is the best thing about *[Mosaic Care]*? And the worst, if any?

Is there anything I should have asked you about your experience with *[Mosaic Care]* which I have missed? Thank you for your time.

Please note: This guide indicates topics we aim to cover in the interviews and some illustrative questions and prompts on how these topics might be approached, rather than a scripted interview schedule that will be universally followed. Given that these are semi-structured interviews, how these topics will be brought into the conversation will be flexible and responsive to interviewee cues. This is owed to the sensitive nature of the topic; we will be guided by participants in their willingness to disclose and reflect on their own experiences with mental health services.

Appendix B

Service Providers - Interview Guide

The purpose of the interview is to learn about your experience as staff member or external partner with *[Mosaic Care]*.

1. To start, tell me a bit about your role at or relationship with *[Mosaic Care]*?

PROBES: Since when?

2. Can you describe the communities to whom *[Mosaic Care]* caters? What are their most pressing concerns? PROBES: Eligibility criteria (e.g., age, catchment, clinical criteria)? Are there prospective clients who reach out and would be eligible but cannot access *[Mosaic Care]* services? Why?

3. Thinking about the delivery of *[Mosaic Care]'s* services:

a. What is your understanding of the *[Mosaic Care]* services provided to clients? What makes *[Mosaic Care]* different from other Mental Health services? PROBES: Purpose? Practice principles? Values?

b. Which services does *[Mosaic Care]* offer? What do they involve? PROBES: clinic? hotline?

c. Were these support services implemented as planned? PROBES: Which support services were adjusted over time? Can you talk more about how the implementation changed? Why were these changes made?

d. What worked well? PROBES: For which support services? Does your program work better for some communities or clients compared to others?

e. What are the challenges you experienced providing services to *[Mosaic Care]'s* clients? PROBES: Interest from the community; buy-in from partners; client retention; resources (e.g., staff), reporting ...?

4. Thinking about recruiting clients into *[Mosaic Care]'s* services:

a. What strategies have you used to recruit clients? PROBES: What strategies have not/worked well?

b. Are you aware of any barriers to accessing services? PROBES: If yes, can you elaborate? What strategies do you use to overcome any challenges for clients to use *[Mosaic Care]'s* services? What additional supports do you need to better recruit and engage with clients?

5. Now, let's think about the reception of *[Mosaic Care]* services in the communities they address:

a. Do you hear feedback from clients using your services? PROBES: In what ways do you gather feedback? How do you respond to feedback (e.g., make changes to services, provide additional resources, referrals, etc.)?

b. Can you share some of the feedback about *[Mosaic Care]*, under what circumstances are these services working? PROBES: Feedback from clients; partners; community stakeholders, etc.

c. How have *[Mosaic Care's]* services impacted your clients? PROBES: Can you give us one or two examples?

6. How does your team ensure consistency in *[Mosaic Care]* service delivery? PROBES: Communication or collaboration with partners? Best practice principles and guidelines?

7. If you could change one thing in regards to how *[Mosaic Care]* is delivered, what would that be?

Is there anything I should have asked you about your experience with *[Mosaic Care]* which I have missed? Thank you for your time.

Appendix C

Service Providers – Group Interview Guide

The purpose of the group interview is to learn about your experience as staff members with *[Mosaic Care]*.

1. To start, can you briefly introduce yourselves? What is your role at *[Mosaic Care]*? Since when?

2. Can you describe the communities to whom *[Mosaic Care]* caters? What are their most pressing concerns? PROBES: Eligibility criteria (e.g., age, catchment, clinical criteria)? Are there prospective clients who reach out and would be eligible but cannot access *[Mosaic Care]* services? Why?

3. Thinking about the delivery of *[Mosaic Care]'s* services:

a. What is your understanding of the *[Mosaic Care]'s* services provided to clients? What makes *[Mosaic Care]* different from other Mental Health services? PROBES: Purpose? Practice principles? Values?

b. Which services does *[Mosaic Care]* offer? What do they involve? PROBES: clinic? hotline?

c. Were these support services implemented as planned? PROBES: Which support services were adjusted over time? Can you talk more about how the implementation changed? Why were these changes made?

d. What worked well? PROBES: For which support services? Does your program work better for some communities or clients compared to others?

e. What are the challenges you experienced providing services to *[Mosaic Care]'s* clients? PROBES: Interest from the community; buy-in from partners; client retention; resources (e.g., staff), reporting ...?

4. Thinking about recruiting clients into *[Mosaic Care]*:

a. What strategies have you used to recruit clients? PROBES: What strategies have not/worked well?

b. Are you aware of any barriers to accessing services? PROBES: If yes, can you elaborate? What strategies do you use to overcome any challenges for clients to use *[Mosaic Care]'s* services? What additional supports do you need to better recruit and engage with clients?

5. Now, let's think about the reception of *[Mosaic Care]* in the communities they address:

a. Do you hear feedback from clients using your services? PROBES: In what ways do you gather feedback? How do you respond to feedback (e.g., make changes to services, provide additional resources, referrals, etc.)?

b. Can you share some of the feedback about *[Mosaic Care]*, under what circumstances are these services working? PROBES: Feedback from clients; partners; community stakeholders, etc.

c. How have *[Mosaic Care]'s* services impacted your clients? PROBES: Can you give us one or two examples?

6. How does your team ensure consistency in *[Mosaic Care]* service delivery? PROBES: Communication or collaboration with partners? Best practice principles and guidelines?

7. If you could change one thing in regards to how *[Mosaic Care]* is delivered, what would that be?

Is there anything I should have asked you about your experiences with *[Mosaic Care]* which I have missed? Thank you for your time.
